# DuoStim – a reproducible strategy to obtain more oocytes and competent embryos in a short time-frame aimed at fertility preservation and IVF purposes. A systematic review

**DOI:** 10.1080/03009734.2020.1734694

**Published:** 2020-04-25

**Authors:** Alberto Vaiarelli, Danilo Cimadomo, Cecilia Petriglia, Alessandro Conforti, Carlo Alviggi, Nicolò Ubaldi, Sergio Ledda, Susanna Ferrero, Laura Rienzi, Filippo Maria Ubaldi

**Affiliations:** a Clinica Valle Giulia, G.EN.E.R.A. Centers for Reproductive Medicine, Rome, Italy;; b Department of Obstetrics and Gynecology, University of Cagliari, Policlinico Universitario Duilio Casula, Monserrato, Cagliari, Italy;; c Department of Neuroscience, Reproductive Science and Odontostomatology, University of Naples Federico II, Naples, Italy;; d Catholic University of the Sacred Heart, Rome, Italy;; e Department of Veterinary Medicine, University of Sassari, Sassari, Italy

**Keywords:** Advanced maternal age, Bologna criteria, double ovarian stimulation, fertility preservation, DuoStim, PGT, poor-prognosis patients, poor-responder patients, reduced ovarian reserve

## Abstract

Recent evidence suggests that follicular development occurs in a wave-like model during the ovarian cycle, where up to three cohorts of follicles are recruited to complete folliculogenesis. This understanding overtakes the previous dogma stating that follicles grow only during the follicular phase of the menstrual cycle. Therefore, in *in vitro* fertilization (IVF), novel protocols regarding ovarian stimulation have been theorized based on the use of gonadotrophins to prompt the growth of antral follicles at any stage of the menstrual cycle. These unconventional protocols for ovarian stimulation aim at a more efficient management of poor-prognosis patients, otherwise exposed to conflicting outcomes after conventional approaches. DuoStim appears among these unconventional stimulation protocols as one of the most promising. It combines two consecutive stimulations in the follicular and luteal phases of the same ovarian cycle, aimed at increasing the number of oocytes retrieved and embryos produced in the short time-frame. This protocol has been suggested for the treatment of all conditions requiring a maximal and urgent exploitation of the ovarian reserve, such as oncological patients and poor responders at an advanced maternal age. At present, data from independent studies have outlined the consistency and reproducibility of this approach, which might also reduce the drop-out between consecutive failed IVF cycles in poor-prognosis patients. However, the protocol must be standardized, and more robust studies and cost-benefit analyses are needed to highlight the true clinical pros and cons deriving from DuoStim implementation in IVF.

## Introduction

Innovations implemented in *in vitro* fertilization (IVF) such as blastocyst culture, single embryo transfer (SET), cryopreservation, and preimplantation genetic testing for aneuploidies (PGT-A) represent important advances in our clinical practice for the management of infertile couples (1). The cryopreservation of gametes and embryos in particular has become fundamental in each treatment, the influence of which has been further boosted by the safety and efficiency of vitrification protocols. In fact, this method ensures higher cryo-survival rates compared with slow-freezing at any stage of preimplantation development (2). All efforts invested in refining IVF during the last decades have aimed at improving its efficacy (number of children born per intention-to-treat) and efficiency (time, drop-out, and risks related to each treatment). With regard to this, an individualized approach (according to each couple’s specific characteristics) has become pivotal for many IVF specialists. If, on the one hand, patients with expected high or normal response to the ovarian stimulation might benefit from validated and reproducible strategies, on the other, the management of poor-prognosis patients is still challenging (3,4). This latter category embraces both advanced maternal age and poor-responder patients. The assessment of the predicted response to controlled ovarian stimulation (COS) is therefore crucial for the personalization of the treatment and to accurately estimate chances of success and inherent risks in addition to complications. Currently, the tailoring of COS is based on: (i) different daily doses and type of gonadotrophins; (ii) the use of GnRH antagonists or agonists to inhibit the luteinizing hormone (LH) peak; (iii) the kind of medications chosen to trigger final oocyte maturation (hGC or GnRH agonist); (iv) the application of fresh or cryopreserved embryo transfer (ET) policy; and (v) whether embryo selection is conducted through PGT-A or solely morphological/morphokinetic criteria. Nevertheless, although several strategies have been proposed, aiming at a maximization of ovarian response and success in poor-prognosis patients (in particular poor responders), no standard management has yet been outlined for them.

The evidence that multiple follicular waves can arise during a single ovarian cycle in humans (5) represented a novel model to describe human folliculogenesis and paved the way to the introduction of unconventional stimulation protocols to manage specific groups of infertile patients (6). The extreme dynamism of folliculogenesis overtakes the classic theory in which a single cohort of follicles starts growing after luteal regression. Today, two more theories have been proposed: the first states that the follicles start growing and regress continuously during the ovarian cycle, and the second states that 2–3 cohorts of antral follicles are recruited in a single ovarian cycle according to the duration of the ovarian cycle. These theories supported the definition of four unconventional protocols for ovarian stimulation: (a) *Random start*, in which COS is started at any phase of the ovarian cycle, a regimen common for fertility preservation purposes to minimize the time invested before starting oncological treatment; (b) *Late follicular phase stimulation* (FPS) in which the stimulation starts after the selection of the dominant follicle or immediately before ovulation in case of fertility preservation; (c) *Luteal phase stimulation* (LPS) in which COS begins with gonadotrophin administration between the 17th and the 21st day of the cycle, a strategy which has been proposed to patients with reduced ovarian reserve or previous cancellation due to no response; (d) *Double stimulation in the same ovarian cycle* (DuoStim) which complements FPS with LPS in the same ovarian cycle, a strategy which has been proposed to poor-prognosis patients, especially due to reduced ovarian reserve and advanced maternal age, but also for fertility preservation purposes (7,8).

DuoStim is useful to all patients who might benefit from increasing the number of oocytes retrieved to maximize the cumulative live birth rate (CLBR) per intention-to-treat (ITT) (9), that is, the current measure of success in IVF (10). Likewise, DuoStim seems to reduce the time to obtain euploid blastocysts and, as Bosch and colleagues suggested, avoid treatment discontinuation (11). The aim of this systematic review is to summarize the evidence already published on putative advantages and disadvantages of the DuoStim protocol for fertility preservation and IVF purposes.

## A glimpse of double stimulation in animal models

Ovarian follicular dynamics have been described in different large-animal models by daily transrectal ultrasound (12–14). Along the inter-ovulatory interval, which varies according to the species (ranging from 17 to 28 days), 2–4 waves of follicular growth might emerge during both the follicular and luteal phase throughout the ovarian cycle. For instance, folliculogenesis in bovine has been extensively studied. A wave of follicular recruitment in cattle is characterized by the synchronous growth of several follicles followed by the selection of dominant ones and the consequent regression of the subordinates. During the oestrus cycle, usually two waves start on day 0 and day 10, but also three waves might be detected on day 0, day 9, and day 16. In the last decades, many authors started to routinely collect oocytes *in vivo* from bovine (once or twice a week). These oocyte retrievals can occur in the presence of the corpus luteum (CL) that in this species covers a large part of the oestrus cycle (16–17 days) (15–17). Even in the luteal phase, the follicles are sensitive to exogenous gonadotrophins that determine an increase in their size and, if the CL is lysed through prostaglandin administration, ovulation can occur as well, and the oocytes might even undergo regular fertilization. Many studies were therefore successfully carried out also *in vitro* and highlighted the fact that oocytes retrieved after LPS might result in good-quality blastocysts and viable offspring after assisted reproductive technology (ART) (18). Also, in the horse, it is well known that follicles respond to gonadotrophins and are selected in the presence of CL. Specifically, during the early gestation in mares (35–45 days post-fertilization), a 4–8-follicle wave starts to grow, stimulated by the secretion of equine chorionic gonadotrophin (eCG) produced by the endometrial cups (an early formation of the chorion placenta). As a consequence of the double activity of follicle-stimulating hormone (FSH) and LH, the CG not only induces the follicular growth but also determines their luteinization, resulting in the formation of accessory CL. It is therefore clear that, in both cattle and horses, the follicles remain sensitive to gonadotrophins even though they are growing under high and/or long-lasting progesterone influence. Moreover, no alteration of the follicular dynamics (e.g. no extension or alteration of the oestrus cycle and no ovarian pathologies) has been observed when the animals were left following their regular reproductive activity after several consecutive ovarian pick-ups (OPU), including those resulting from LPS (19). Of note, in cattle, numerous consecutive OPUs are possible only thanks to the ablation of dominant follicles that would otherwise have negatively affected the recruitment of the new cohorts of antral follicles.

Interestingly, the presence of CL and high levels of progesterone seems to modulate the effect of gonadotropins. For instance, in sheep, progesterone has been proposed to be a key endocrine signal governing periodic increases in both serum FSH concentrations and number of follicular waves per cycle. Whether these effects of luteal progesterone on antral follicle lifespan are local, systemic (i.e. mediated by changes in FSH/LH secretion), or both remains to be elucidated. Hence, under the influence of luteal progesterone, the sensitivity of FSH-producing gonadotrophins to GnRH may increase, resulting in a higher secretion of FSH from the pituitary gland. Moreover, circulating progesterone concentrations may dictate the clearance rate of circulating FSH (14).

## When is DuoStim indicated?

Oocyte cryopreservation is a great challenge for oncological patients urgently needing fertility preservation prior to undergoing chemotherapy and/or radiotherapy (20). In these patients, it is crucial to maximize the number of cryopreserved oocytes after COS in the short time-frame, to increase the chance of future conception(s). In this regard, an ideal number of oocytes to cryopreserve can be considered to be at least 10–15, mainly depending on maternal age (20–22). For this reason, a random start protocol is used to speed up fertility preservation and therefore reduce the delay to cancer treatment. Such a protocol is possible since there is no need for ovarian–endometrial synchrony. However, in many patients, there are not enough oocytes collected to ensure reasonably good chances of pregnancy. Based on this, when the time is limited and the oocytes collected from one stimulation are insufficient, DuoStim protocols might be discussed with the oncologist together with the patients as a valuable option for fertility preservation (23–26).

DuoStim, combining two consecutive stimulations spanning a 5-day interval, has been put forward as a valuable opportunity also for the management of poor-prognosis patients such as women with reduced ovarian reserve and/or advanced maternal age. The aim then is to maximize the number of oocytes retrieved in a single ovarian cycle, or to rescue patients in whom no oocytes were retrieved or competent embryos were not produced after conventional FPS (27–30). For these thorny populations of patients, there is insufficient evidence to outline an ideal management since, regardless of the COS protocol adopted, consistently low live birth rates have been reported. In fact, oocyte quantity and quality, which are both critical to increase CLBR per ITT, could have suffered a dramatic physiological decline in these women. Of note, if ageing impairs oocyte competence due to insults such as mitochondria and cohesion dysfunction, shortening of the telomeres, and spindle instability (31), also ‘young’ oocytes suffer from impairments that shape the window of the woman’s fertility. Specifically, the oocyte aneuploidy rate follows a U-shaped curve with its highest prevalence before menarche and just before menopause, and its lowest prevalence at the age of 25 years (32). Conversely, the oocyte competence to develop as a blastocyst seems constant across the age range of the woman until the age of 40 years (33), when it abruptly decreases. Both these curves outline a sharp increase in the aneuploid blastocyst rate, which from a rate of 25–30% in women younger than 35 years might reach rates higher than 90% in patients older than 42 years of age (34,35).

These data affect also the choice of an effective COS strategy depending on the age range of the patient (<35 y, 35–40 y, or >40 y) (36). Moreover, beyond being responsible for a decreased fertility, aneuploidies cause an increased prevalence of vital chromosomal abnormalities, increased miscarriage rates, as well as an increased prevalence of numerical chromosomal abnormalities in new-borns (31). Nevertheless, no therapy is available at present to minimize the ageing-related damage listed above. The only available strategy is to compensate the physiological decline in oocyte and embryonic competence by collecting the highest possible number of mature oocytes (36,37). In our setting, the DuoStim protocol is always combined with PGT-A and single vitrified–warmed euploid blastocyst transfer, independently of the number and morphological quality of the embryos obtained after the two stimulations (38). The aim of this approach is to try to reduce both the frustrating reiterated implantation failures and the miscarriage rate after IVF (39,40). These aspects are especially crucial to reduce the drop-out in poor-prognosis patients (as for instance the patient fulfilling the Bologna criteria) (41) without compromising the overall efficacy of treatment (42).

## Performance of the DuoStim protocol

The DuoStim protocol entails two consecutive stimulations in a single ovarian cycle with the intent to increase the number of oocytes retrieved and the blastocysts available for transfer or PGT. The protocol involves a pre-treatment with luteal oestradiol priming (4 mg/day of oestradiol valerate) on day 21 of the previous menstrual cycle to promote the synchronization and coordination of the follicular growth (43). Transvaginal ultrasound and basal assessment of the ovaries are performed on day 2 to day 3 of the menstrual cycle, then luteal oestradiol priming is stopped, and FPS is started with a fixed dose of recombinant FSH (r-FSH) 300 IU/day plus r-LH 150 IU/day for 4 days. Follicular growth is monitored on day 5 and then every 2–3 days depending on the progress of the ovarian response. Flexible GnRH antagonist is administered daily after the identification of a leading follicle with a diameter ≥13–14 mm in FPS and LPS until the day of ovulation trigger. The final maturation of oocytes is triggered with a subcutaneous bolus of buserelin (dose 0.5 ml) to reduce the time of luteolysis (44). Egg retrieval is performed 35 h after the trigger. Five days after the first retrieval, LPS is started with the same protocol and daily dose regardless of the number of visible antral follicles. In our group we propose DuoStim protocol combined with PGT-A and single euploid vitrified–warmed blastocyst transfer.

## Search procedure

This systematic review was conducted by searching the MEDLINE (PubMed), Scopus, and Embase databases up to October 2019. Combinations of the following keywords and search terms were used: ‘DuoStim’, ‘luteal phase stimulation’, ‘luteal phase ovarian stimulation’, ‘dual stimulation’, ‘double stimulation’, ‘ovarian stimulation’, ‘assisted reproductive technique’, ‘*in vitro* fertilization’. No time or language restriction was adopted, and queries were limited to human studies. The reference lists of relevant reviews and articles in press were also hand-searched. Three reviewers (AV, DC, AC) evaluated titles and abstracts. Duplicates were removed using Endnote online software and manually. Disagreements were discussed and ultimately resolved by consensus between all authors with the involvement of the most experienced ones (CA, LR, FMU). We included all studies published that comprised case series and case reports in which two consecutive stimulations were performed in the same menstrual cycle in infertile women undergoing IVF or fertility preservation programmes.

**Figure 1.**
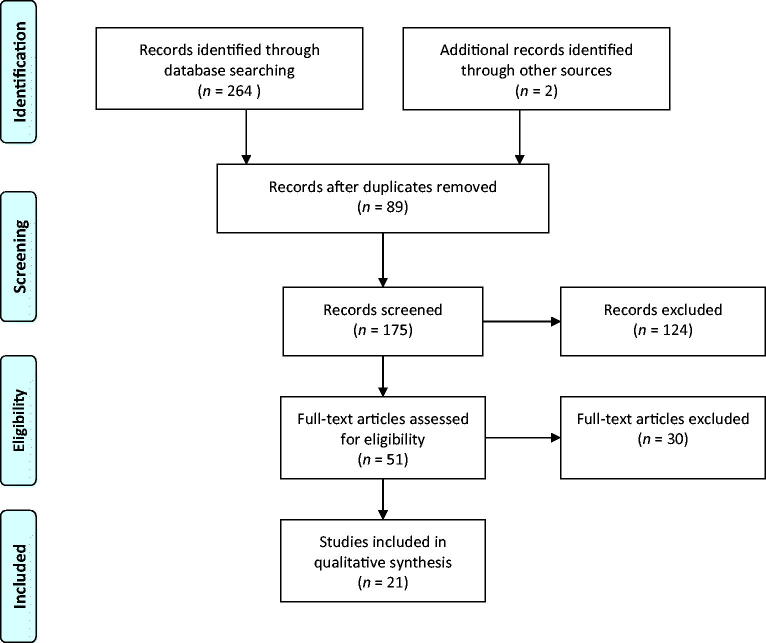
PRISMA 2009 flow diagram.

A total of 264 search items were identified. After removal of duplicates a total of 175 papers were scrutinised. Fifty-one papers were assessed for eligibility. In the following review, we included 21 papers, while 30 were excluded because they were reviews, abstracts, or studies comparing FPS and LPS not in the same patient or ovarian cycle (Figure 1). Table 1 represents a summary of all the studies included in this systematic review and is presented as an electronical supplement.

**Table 1.** Summary of the clinical evidence produced via double stimulation in the same ovarian cycle to date.

Study	Design	Inclusion criteria	Number of patients	FPS protocol	Trigger	LPS protocol	To avoid the LH surge	Conclusions
Xu and Li (45)	Case report	Poor-responder patient 41 y old	1	CC (50–100 mg/day) + FSH 150 IU/day	GnRH-a (triptorelin 0.2 mg) and hCG 10,000 IU	CC (50–100 mg/day) + FSH 150 IU/day	Ibuprofen	LPS might rescue unsuccessful oocyte retrievals after FPS
Kuang et al. (8)	Pilot	AFC ≤6; ≤5 oocytes retrieved from a previous cycle; history of ovarian surgery; FSH level: 10–19 IU/L; maternal age ≥40 y	38	CC 25 mg/day; LE 2.5 mg/day (4 days); HMG 150 IU/day	GnRH-a 0.5 mg	LE 2.5 mg/day + HMG 225 IU/day	Ibuprofen 0.6 g + MPA	Higher chance to retrieve oocytes in a single ovarian cycle
Moffat et al. (24)	Commentary	Fertility preservation and conditions requiring larger cohorts of oocytes	NR	r-FSH 300 IU/day	GnRH-a (triptorelin 0.2 mg)	r-FSH 300 IU/day	GnRH-ant	Increased number of oocytes collected in less than 30 days with a patient-friendly approach
Ubaldi et al. (38)	Proof of concept	AMH ≤1.5 ng/mL; AFC ≤6; ≤5 oocytes retrieved from a previous cycle; maternal age ≥35 y	51	r-FSH 300 IU/day + r-LH 75 IU/day	GnRH-a (buserelin 50 IU)	r-FSH 300 IU/day + r-LH 75 IU/day	GnRH-ant	LPS increased the number of patients producing at least 1 euploid blastocyst and undergoing a transfer in a single ovarian cycle
Wei et al. (46)	Retrospective	Maternal age >40 y; prior history of poor response; ≤3 oocytes retrieved; AFC <6.	23	CC 50 mg/day + LE 2.5 mg/day (5 days) + HMG 150 IU/day	GnRH-a 0.1 mg (FPS) and hCG 10,000 IU (LPS)	CC 50 mg/day + LE 2.5 mg/day (5 days) + HMG 150 IU/day	MPA	More oocytes collected after LPS with respect to FPS
Zhang et al. (47)	Retrospective	POR defined according to the Bologna criteria	153	CC 50 mg/day + HP-FSH 150 IU/day	GnRH-a (triptorelin 0.2 mg)	CC 50 mg/day + HP-FSH 150–225 IU/day	Ibuprofen 300 mg every 6 h from GnRH-a injection to the day of follicle aspiration	LPS results in more COC, MII, and zygotes; LPS-derived embryos resulted in higher IR
Tsampras et al. (23)	Pilot	Fertility preservation in oncological patients	10 oncological patients	HMG 150–450 IU/day	hCG 5000 IU	HMG 150–450 IU/day	GnRH-ant	Increased number of oocytes vitrified after COS with no delay in starting chemotherapy
Vaiarelli et al. (7)	Observational	AMH ≤1.5 ng/mL; AFC ≤6; ≤5 oocytes retrieved from a previous cycle; maternal age ≥35 y	128	r-FSH 300 IU/day + r-LH 75–150 IU/day	GnRH-a (buserelin 50 IU)	r-FSH 300 IU/day + r-LH 75–150 IU/day	GnRH-ant	No difference in the embryological outcomes between FPS and LPS (fertilization, blastulation, and euploidy rates)
Cardoso et al. (48)	Retrospective	Previously failed IVF treatment(s)	13	r-FSH 225 IU/day + HMG 75 IU/day	GnRH-a (triptorelin 0.2 mg)	r-FSH 225 IU/day + HMG 75 IU/day	GnRH-ant	Higher number of oocytes in a single ovarian cycle
Liu et al. (49)	Case-control	Women ≥38 y	116	r-FSH 150–300 IU/day + r-LH 75–150 IU/day	r-hCG 250 mg	HMG 225 IU/day	Long agonist (13pz); Short agonist (27pz); GnRH-ant (53pz); MPA (23pz)^a^	Higher number of oocytes in a single ovarian cycle
Cimadomo et al. (52)	Paired case-control study	AMH ≤1.5 ng/mL; AFC ≤6; ≤5 oocytes retrieved from a previous cycle; maternal age ≥35 y	188	r-FSH 300 IU/day + r-LH 75–150 IU/day	GnRH-a (buserelin 50 IU)	r-FSH 300 IU/day r-LH 75–150 IU/day	GnRH-ant	LPS generates larger cohorts of oocytes with comparable developmental and chromosomal competence than paired-FPS-derived ones
Zhang et al. (74)	Retrospective	Poor responders fulfilling the Bologna criteria^b^	61	CC 50–100 IU/day (5 days) + HMG 75–150 IU/day	r-hCG 250 mg	CC 50–100 IU/day (5 days) + HMG 75–150 IU/day	Dufaston	More oocytes retrieved but lower MII rate after LPS than after FPS; similar LBR and CPR
Rashtian and Zhang (51)	Retrospective	FSH level >15 IU/mL; AFC 1–8; 1 previous failed conventional IVF cycle	69	CC 50 mg/day + LE 2.5 mg/day (5 days) + FSH 75 IU/day	GnRH-a (FPS)/r-hCG (LPS)	CC 50 mg/day + LE 2.5 mg/day (5 days) + FSH 75 IU/day	GnRH-ant	No difference in the number of oocytes retrieved between FPS and LPS
Madani et al. (53)	Prospective	Poor responders fulfilling the Bologna criteria^b^	104	CC 25 mg/day + LE 2.5 mg/day (4 days) + HMG 150 IU/day	GnRH-a	LE 2.5 mg/day + HMG 225 IU/day	Ibuprofen 0.6 g + MPA	Fertilization rate and number of frozen embryos higher after FPS than LPS; double stimulation is a time-saving and patient-friendly regimen
Jin et al. (50)	Retrospective	Poor responders according to Bologna criteria^b^	76	CC 50 mg/day + LE 100 mg/day or 5 mg/day (5 days) + HMG 150–300 IU/day	GnRH-a (triptorelin 0.1 mg) or hCG 5000–10,000 IU	CC 50–100 mg/day + HMG 150–300 IU/day	GnRH-ant	More oocytes and embryos obtained, as well as lower cancellation rate within an ovarian cycle with double stimulation
Vaiarelli et al. (27)	Multicenter observational	AMH ≤1.5 ng/mL; AFC ≤6; ≤5 oocytes retrieved from a previous cycle; maternal age ≥35 y	310	r-FSH 300 IU/day + r-LH 150 IU/day	GnRH-a (buserelin 50 IU)	r-FSH 300 IU/day + r-LH 150 IU/day	GnRH-ant	Higher rate of patients obtaining at least 1 euploid blastocyst in a single ovarian cycle thanks to the contribution of LPS
Sighinolfi et al. (25)	Commentary	Fertility preservation in oncological patients	NR	r-FSH 200 IU/day or HMG 200 IU/day	GnRH-a	r-FSH 300 IU/day + r-LH 75 IU/day	GnRH-ant	Increased number of oocytes collected and vitrified in a short time-frame
Lin et al. (75)	Pilot	Poor responders fulfilling the Bologna criteria^b^	60	HMG 225 IU/day + CC 100 IU/day	r-hCG + GnRH-ant	r-FSH 300 IU/day + r-LH 150 IU/day	MPA 10 mg	More oocytes and day-3 embryos after LPS than after FPS
Hatirnaz et al. (55)	Retrospective study	POI^c^	51	Single dose of r-FSH 225 IU the day of trigger	hCG 10,000 IU	LE 5 mg/day + single dose of r-FSH 225 IU the day of trigger	hCG 10,000 IU	Dual stimulation compared to subsequent conventional stimulations reduced the number of oocyte retrievals performed to obtain at least 2 cleavage stage embryos
Alsbjerg et al. (54)	Case series	Poor responders fulfilling the Bologna criteria (<42 y)^b^	54	Corifollitropin-alfa + r-FSH 300–375 IU/day OR r-FSH 300 IU/day + r-LH 150 IU/day	GnRH-a (FPS) or hCG (LPS)	Corifollitropin-alfa + r-FSH 300–375 IU/day OR r-FSH 300 IU/day + r-LH 150 IU/day	GnRH-ant	More oocytes retrieved after LPS; lower cancellation rate after double stimulation
Vaiarelli et al. (42)	Case series	Poor responders fulfilling the Bologna criteria^b^	100 patients choosing DuoStim versus 194 choosing conventional COS	r-FSH 300 IU/day + r-LH 150 IU/day	GnRH-a (buserelin 50 IU)	r-FSH 300 IU/day + r-LH 150 IU/day	GnRH-ant	DuoStim prevents drop-out after a first failed attempt, thereby increasing the CLBR per ITT

^a^ Long agonist: controlled ovarian stimulation protocol with long GnRH agonist; short agonist: controlled ovarian stimulation protocol with short GnRH agonist.

^b^ Poor responders (fulfilling Bologna criteria): according to the Bologna criteria the patients should have at least two of these characteristics: (i) maternal age (≥40 years); (ii) a previous ovarian response ≤3 oocytes with a conventional stimulation protocol; (iii) an abnormal ovarian reserve test (i.e., AFC 5–7 follicles or AMH 0.5–1.1 ng/mL).

^c^ Premature ovarian insufficiency (POI) defined as FSH levels higher than 40 IU/L, up to two ovarian follicles (2–9 mm) at the baseline pelvic scan, presence of oligomenorrhea/amenorrhea, and low levels of AMH <0.30 pg/mL.

AFC: antral follicle count; AMH: anti-Müllerian hormone; CC: clomiphene citrate; CLBR: cumulative LBR; COC: cumulus oocyte complex; COS: controlled ovarian stimulation; CPR: cumulative pregnancy; FPS: follicular phase stimulation; FSH: follicle-stimulating hormone; GN : gonadotrophin; GnRH-a: GnRH agonist trigger; GnRH-ant: controlled ovarian stimulation protocol with antagonist protocol; hCG: human chorionic gonadotrophin; HMG: human menopausal gonadotrophin; HP-FSH: highly purified FSH; IR: implantation rate; ITT: intention-to-treat; IVF: *in vitro* fertilisation; LBR: live birth rate; LE: letrozole; LH: luteinizing hormone; LPS: luteal phase stimulation; MII: metaphase II; MPA: medroxyprogesterone acetate; NR: not reported; POI: premature ovarian insufficiency; r-FSH: recombinant FSH; r-hCG: recombinant hCG; r-LH: recombinant LH.

## Updated body of evidence regarding the clinical implementation of DuoStim

The first case report of LPS subsequent to FPS was published in 2013 by Xu and Li. They conducted LPS in a 41-year-old woman in whom no eggs were retrieved from a first oocyte pick-up (45). Since then, several studies have been published of different design and number of patients undergoing double stimulation for either fertility preservation or IVF purposes. In 2014, Kuang et al. (8) reported more opportunities for retrieving oocytes in a single month thanks to what is known as the ‘Shanghai protocol’. According to this protocol, the first stimulation was a conventional FPS, whereas LPS started on the subsequent day of the first oocyte retrieval, when two or more antral follicles were identified. Regarding these two stimulations, two different regimens were adopted: for FPS, a combination of clomiphene citrate 25 mg/day starting on day 3 of the cycle and until the triggering of ovulation, letrozole 2.5 mg/day starting on day 3 for a total of 4 days, and human menopausal gonadotrophin (HMG) 150 IU/day starting on day 6 and until the triggering of ovulation; for LPS, letrozole 2.5 mg/day and HMG 225 IU/day, both starting from the day of first oocyte retrieval and until the second triggering of ovulation. Medrossiprogesterone acetate (MPA) was also administered at the end. For both stimulations, ovulation was triggered with triptorelin 0.1 ml when follicular maturation was finally achieved (8). In 2016, Wei et al. (46) confirmed the same results as those of Kuang et al., with the same protocol adopted in patients aged >40 y, with a prior history of poor response defined as ≤3 oocytes retrieved and an antral follicle count (AFC) <6.

In the same year, Zhang et al. (47) showed, in a retrospective study based on 153 patients fulfilling the Bologna criteria, that LPS results in more cumulus–oocyte complexes, metaphase II (MII) oocytes, and zygotes and that embryos obtained after LPS are characterised by higher implantation rates. Moreover, Ubaldi et al. (38) published a proof of concept that defines the first application of DuoStim (GnRH antagonist protocol with a fixed r-FSH 300 IU/day dose combined with r-LH 75 IU/day in both FPS and LPS) in 51 poor-prognosis patients (anti-Müllerian hormone [AMH] < 1.5 ng/mL, AFC <6 follicles, and/or <5 oocytes retrieved in previous IVF cycles) undergoing ICSI and PGT-A. GnRH agonist trigger was then adopted with the aim of reducing the half-life of the CL after oocyte collection and facilitate the recruitment of follicles from the luteal wave. Here, no statistically significant difference was found in terms of number of MII oocytes retrieved, fertilisation, blastocyst, and euploid blastocyst rates between FPS and LPS. As a consequence, DuoStim increased the final transferable blastocyst yield per ovarian cycle with respect to FPS-only (38). These results were confirmed in a larger number of patients one year later (7). In 2017, two retrospective studies reported that double stimulation increases the number of oocytes retrieved in a short period of time: Cardoso et al. (48) compared the conventional antagonist protocol to DuoStim in 13 patients with a previous history of failed IVF treatments, while Liu et al. (49) investigated the efficacy of DuoStim compared with FPS-only in advanced maternal age patients (mean age: 42 ± 3 years). In both cases, twice as many embryos were obtained with DuoStim. In Liu’s study also, the cancellation rate decreased from 37% to 18% with DuoStim.

A further pilot study published in 2017 by Tsampras et al. (23) tested the efficacy of double stimulation for fertility preservation in oncological patients. Ten patients underwent double stimulation with GnRH antagonist and HMG protocol. This protocol increased the number of oocytes retrieved and consequently vitrified, without delaying cancer treatment. In 2018, another two studies (50,51) demonstrated how double stimulation could be effective in patients with poor ovarian reserve. Jin et al. (50) in a retrospective study compared double stimulation (Group A, *n* =  76 poor responders) to LPS-only (Group B) and to mild ovarian stimulation (Group C). Although after FPS fewer oocytes were collected and fewer embryos produced in Group A than in Groups B and C, their overall numbers in a single ovarian cycle were significantly higher with the contribution of LPS (50). Rashtian and Zhang assessed whether DuoStim in advanced maternal age patients (mean age: 42 years) might produce a higher number of oocytes compared with FPS-only. In their study, 69 women with diminished ovarian response underwent a GnRH antagonist protocol with r-FSH, letrozole, and clomiphene citrate for both stimulations. The ovulation was triggered with GnRH agonist in FPS and with hCG in LPS. There was no statistically significant difference between the number of oocytes retrieved. Therefore, the addition of LPS to FPS doubled the number of inseminated oocytes in a single ovarian cycle (51).

Two more studies were published in 2018: Cimadomo et al. (52) reported that the cohorts of oocytes obtained after LPS from 188 patients are larger than their paired-FPS-derived cohorts and showed a comparable competence in terms of blastulation rate and euploidy rate. Vaiarelli et al. (27), on the other hand, conducted a multicentre study which confirmed the reproducibility of the results with consistently superior outcomes utilising the DuoStim application in 310 poor-prognosis patients from four IVF centres. In particular, 65.5% of the patients obtained at least one euploid blastocyst after DuoStim rather than 42% if only FPS had been carried out. In 2019, Madani et al. (53) published a prospective clinical study based on the adoption of DuoStim to treat 121 patients fulfilling the Bologna criteria. Double stimulation was performed by letrozole, clomid, HMG, and GnRH agonist and was found to be a time-saving and patient-friendly regimen. Alsbjerg et al. (54) reported a case series of 54 poor responders classified according to the Bologna criteria (mean age: 37 years), who were treated with DuoStim performed with corifollitropin-alfa. Also in this study, DuoStim was confirmed as a valuable alternative to conventional FPS to increase the overall number of oocytes retrieved and decrease the risk for cycle cancellation. Hatirnaz et al. (55) demonstrated in a retrospective study including 51 women that double stimulation is convenient in the management of patients with premature ovarian insufficiency (POI). They reported that double stimulation halves the number of oocyte retrievals required to obtain at least two transferable good-quality cleavage stage embryos compared with several consecutive conventional stimulations. In a recent observational study of patients fulfilling the Bologna criteria, 100 out of 297 patients had agreed to undergo DuoStim after extensive counselling (42). In these couples, the CLBR per ITT was 15% in a single ovarian cycle, whereas the corresponding figure was 8% among the 197 patients choosing a conventional COS strategy and undergoing up to two oocyte retrievals in a 2-year period. In fact, only 17 patients not conceiving after a first failed attempt returned for a second one in the latter study arm. Therefore, the authors underlined that DuoStim application in patients fulfilling the Bologna criteria prevents cycle discontinuation, thereby conferring a higher chance to conceive in a shorter time-frame (42). Furthermore, Cimadomo et al. (56) identified the miRNomic signatures of the follicular fluids collected from 15 poor-prognosis patients after FPS and paired-LPS. No difference was reported, thereby further suggesting the safety of LPS. Lastly, an ongoing non-selection study, the interim analysis of which has been presented at the ESHRE annual meeting held in Barcelona in 2018 (57), outlined the absence of differences in terms of obstetrical and neonatal outcomes between FPS-derived and LPS-derived live births.

## Weaknesses, risks, and concerns related to DuoStim

The personalisation of COS has represented a game-changer for the management of poor-prognosis patients undergoing IVF (29). Clearly, DuoStim fits into this scenario, and its strengths and weaknesses must therefore be outlined. DuoStim still needs a cost-benefit analysis and a randomised controlled trial (RCT) that compares it with consecutive conventional FPS. More data regarding its safety from a biological, clinical, and neonatal perspective are still required. The mandatory need for a freeze-all strategy represents an inevitable limitation (27). Then, a consensus regarding DuoStim protocol should be built in terms of timing, kind, and dosage of the medications adopted in the LPS. The luteal phase is characterised by the presence of the CL, higher progesterone and oestrogen levels, and evidence demonstrating that, after GnRH agonist trigger in antagonist protocols, luteolysis differs greatly among patients and depends on: (i) levels of progesterone on the day of final oocyte maturation and oocyte retrieval; (ii) duration of ovarian stimulation; (iii) number of days of suppression; (iv) total dosage of the medications used for ovarian suppression; and (v) number of oocytes retrieved (44). Therefore, the beginning of the menstruation following agonist triggering is to be considered patient-specific. The use of gonadotropins a few days after agonist trigger in the luteal phase of the ovarian cycle allows the rescue of small antral follicles that otherwise would have been lost because they are recruited from a physiologically anovulatory luteal wave (6,7). Yet, the choice of the medications in a DuoStim protocol should be based on their effectiveness. Although the Shanghai protocol suggested the use of clomiphene citrate and letrozole in the FPS, a Cochrane review published in 2017 did not support their use, also due to an increased risk for cycle cancellation as well as for a lower number of oocytes retrieved in poor responders (58). Similarly, several RCTs (59–62) and meta-analyses (63–65) reported better efficacy in terms of oocyte retrieval after COS with r-FSH compared with HMG. Moreover, the total amount of gonadotrophins used is lower when COS is performed with r-FSH compared with HMG (66,67). All these aspects must be considered when we treat poor-prognosis patients where even a single oocyte can make a huge difference in terms of cumulative results and cost-benefit (28).

Finally, the choice of a r-LH supplementation during DuoStim is based on its role in promoting steroidogenesis and folliculogenesis (68). Indeed, LH increases androgen production, stimulates early stages of follicular growth, increases the recruitment of pre-antral and antral follicles, and increases the expression of FSH receptors in the granulosa cells (69). All these aspects are especially important in patients of advanced maternal age or poor/sub-optimal responders subject to ageing-derived reduction in androgen production and to inadequate levels of endogenous androgens, in turn associated with a decreased ovarian sensitivity and responsiveness to exogenous FSH (29,70,71). Although there is no consensus about LH measurement and a correct therapeutic window, its supplementation in COS seems helpful for the treatment of advanced maternal age patients undergoing a GnRH antagonist protocol in terms of lower dose of r-FSH and higher IVF outcomes with no increase of the overall costs (72,73). All these issues indirectly related with DuoStim are still controversial and debated in the scientific community.

## Conclusions

The wave theory of follicle recruitment was developed in several animal models before it was confirmed in women. That evidence introduced important clinical implications for the personalized approach of COS in specific patients undergoing IVF. The exploitation of both the ovulatory (major) and the anovulatory (minor) waves has allowed the implementation of new unconventional COS protocols, among which DuoStim is one of the most promising, especially for the treatment of poor-prognosis patients (advanced maternal age and/or reduced ovarian response) and patients requiring fertility preservation for medical reasons. Although the quality of the clinical studies focussed on the implementation of DuoStim is moderate–low, all of them highlighted that a double stimulation is a valid option to increase the number of oocytes/embryos in a single ovarian cycle. The LPS-derived cohorts of oocytes were also larger than their paired-FPS-derived ones while showing a comparable competence in terms of blastulation and euploidy rate. These results certainly support the important contribution LPS has in poor-prognosis and oncological patients, with a limited amount of time and insufficient numbers of oocytes to grant a reasonable chance of IVF success. Nevertheless, additional clinical and basic research studies on this topic are needed to further encourage the personalisation of COS in specific populations of patients.
